# Investigating spatial distribution of kindergartens in Chinese cities: A spatial stratified heterogeneity approach

**DOI:** 10.1371/journal.pone.0338737

**Published:** 2026-01-23

**Authors:** Bo Zhang, Shixiong He, Lei Jiang

**Affiliations:** 1 Center for Human Geography and Urban Development, School of Geography and Remote Sensing, Guangzhou University, Guangzhou, China; 2 Guangdong Provincial Center for Urban and Migration Studies, Guangzhou, China; 3 Institute of Economics, Shanghai Academy of Social Sciences, Shanghai, China; International University of Business Agriculture and Technology, BANGLADESH

## Abstract

The spatial equity of kindergarten distribution is a critical concern for China’s educational policy. This study investigates the socioeconomic determinants underlying the spatial stratification of kindergartens across 323 Chinese cities. Utilizing a spatial stratified heterogeneity framework, we employed web-crawling techniques to gather kindergarten location data and applied Geographical Detector (GD) analysis complemented by Pearson correlation and Grey relational degree methods to quantify the influence and interactions of key factors. Our principal results indicate that: (1) The distribution of kindergartens is highly aggregated in affluent eastern coastal regions, but this pattern becomes more scattered when normalized per capita, revealing disparities in access. (2) Economic capacity (per capita income) and educational attainment (average years of education) are the two most powerful single factors (q-statistics of 0.351 and 0.343, respectively) explaining the spatial distribution. (3) Critically, interaction effects between factors—particularly between income and marriageable population or education levels—are shown to be non-linearly enhanced, meaning their combined effect is stronger than the sum of their individual parts. This study’s main contribution is a robust, methodologically novel empirical analysis that moves beyond linear assumptions to identify the synergistic drivers of kindergarten distribution. We conclude that policy makers must prioritize interventional strategies that simultaneously address economic and educational development to effectively reduce spatial inequities in preschool education access. Moreover, regional economic support policies or targeted public investment in kindergartens could bridge the gaps between urban and rural areas and promote more equitable access to preschool education.

## 1. Introduction

This article investigates how the socioeconomic factors determine the number and the distribution of kindergartens in China. In China, the earliest kindergartens were offered and regulated by the workplace (*Danwei* in Chinese; See [1]) in a collective form for their own employees before 1980s. These employer-established kindergartens were the primary form of urban preschool education, offering a crucial service that enabled parental employment and fostered loyalty to the work unit. Whereas the first-round reform of kindergartens occurred during 1980s-2000, when several official regulations and guidelines were introduced; at this time new kindergartens had separated from workplaces including large state-owned enterprises (SOEs), government agencies, universities, and military units [2,3]. After the economic reforms of the 1980s and 1990s, many SOEs were restructured or privatized, leading to the closure or transfer of many of these kindergartens to the public education system. Since the year 2000 there has been growing concern about pre-school education in China, and by 2020, national government funding for preschool education had climbed to 25.32 billion Yuan (3.47 billion USD), five times more than the preschool investment in 2011. The full-day programs serving children between aged 3 and 6 in the kindergarten play a significant role in China’s pre-school education system. However, not all children in China have equal access and affordability to receive a preschool education. In response, the State Council issued the “Outline of the National Medium and Long-Term Education Reform and Development Plan (2010-2020)” in 2010 to promote the equality of preschool education. Consequently, in some poor regions in northwest China the preschool education is even free of charge [2]. According to this plan, all the newly constructed commercial residential communities after 2010 should provide the arrangements for the construction of kindergartens. Moreover, the construction of the kindergartens should be put into the local urban planning in the newly established urban neighborhoods by the local government as a mandatory composition of the public education resources. With the strong backing of the Chinese national government, the number of kindergartens across the country rose from around 150,000 in 2010–297,715 in 2020. Correspondingly, the number of children in kindergarten has increased from 29,767,000–48,052,000, which is approximately 88.1% of children between the ages of 3 and 6. The total number of kindergarten directors and full-time teachers has increased by 2 million, and is now over 3.5 million, 1.3 times higher compared to 2011; furthermore, the student-teacher ratio has dropped from 26:1 in 2011–15:1 in 2021, thus ameliorating the shortage of kindergarten teachers. More importantly, the quality of teachers has improved significantly, with the percentage of directors and full-time teachers with specialist degrees or above reaching 87.8% in 2021, 24 percentage points higher than in 2011.

Despite the remarkable achievements in the past 20 years in terms of preschool education in China, problems remain to be solved. First, as already mentioned, preschool education has been heavily promoted and expanded since 2000, however, concern remains about whether there is an unbalanced development between its access and its quality. In addressing this problem, Li et al. [4] argue that promoting enrollment of the kindergarten alone would be inadequate to fulfill the aim of promoting children’s early development if with low quality of the preschool education offering. In fact, the quality of the preschool education is evidenced to be associated with the socioeconomic development and performance [5]. Second, despite efforts to diminish the spatial heterogeneities in different geographical locations, there are still large differences among populations, cities and regions regarding kindergarten accessibility and affordability. Preschool education is not yet compulsory. Although the State Council introduced a series of policies encouraging non-governmental funds to establish inclusive private kindergartens under the document framework, “Law of the People’s Republic of China on the Promotion of Privately-Run Schools” in 2002, vacancies remain and demands differ across populations and regions. To date, as far as we are aware, no empirical study explores the spatial distribution of the kindergartens at macro level; we therefore aim to analyze the spatial heterogeneity of kindergartens at city level.

Broadly speaking, the two types of kindergartens in terms of its financial sources, are the public and private funded. Data from the Ministry of Education of the People’s Republic of China (MOE) indicates that publicly funded kindergartens in 2018 accounted for 33.25% of the total, while 66.75% of kindergartens were funded privately. Whereas in 2010, 22.6% were publicly funded kindergartens compared to 77.74% private. The growth of publicly funded kindergartens reveals the state’s effort to promote an “affordable preschool education.” The 2018 jointly issued document of the Communist Party of China Central Committee and the State Council, “Deepening Reform and Standardizing Development of Preschool Education,” prioritized “affordable preschool education” in the aim to reduce the number of expensive private kindergartens. By 2021 affordable kindergartens had increased to 83% and accepted 87.8% of all children between 3–6 years old into kindergarten. This proportion is even higher in rural and remote areas, up to 90.6% [6]. To guarantee affordable preschool education means that even these non-public kindergartens need to receive abundant financial support from the government to maintain normal running. Thus, the establishment of kindergartens may be largely reliant on the local financial condition and the strength of the support received. Although not encouraged, approximately 10% of kindergartens still provide preschool education while charging relatively high tuition and do not receive any government funds. These kindergartens are largely aggregated in first and second tier cities such as Beijing, Shanghai, Guangzhou, Nanjing, and Chengdu. In this case, generally speaking, the parents who can afford high tuitions for their kids have a high education level and high income themselves. We can safely assume that the demand for kindergartens varies across demographic regions, which likely contributes to the spatial heterogeneity of kindergartens.

Although proactive measures have been taken by the government at all levels to narrow the gap between rural and urban areas in the past 10 years, significant differences between rural and urban in terms of the quality of preschool education still exist. Uneven economic, societal and educational development between urban and rural areas is also apparent in enrollment rates, teacher quality, condition of infrastructure, and other aspects of the preschool education system [7,8]. Statistics from MOE show that the enrollment rate of the kindergartens has increased markedly from 62.3% in 2011, to 88.1% in 2021. At present, the spatial inequity of the kindergartens is significantly reduced, with 80% of the newly established schools located in the middle and western part of the country, and 60% situated in rural areas. Despite the effort, kindergartens in rural areas are decreasing; where they comprised 50% of the total in 2010, the percentage drops to 35% in 2019, mainly due to the rural-urban migration. There is also a large gap between rural and urban area in terms of quality. Li et al. [4] argue for the necessity to provide a consolidated national base line standard of quality for preschool education which is prioritized and serves rural children. This means that the public funding and other funding sources should be designated to the areas where the quality of preschool education is relatively lower.

The kindergarten is essential for not only the early cognition development and whole lives of individual children, but also be beneficial to the families and the society; however, the spatial distribution of kindergartens at city level in China from a macro perspective is not yet understood. We also acknowledge that socioeconomic factors influence kindergartens in multiple ways. Therefore, based on the perspective of spatial heterogeneity, we ask, 1. What characteristics do the spatial distribution of kindergartens present? 2. What factors impact on the spatial distribution of kindergartens? 3. How do the interaction terms of the socioeconomic factors influence the spatial distribution of kindergartens? We first apply a spatial visualization technology to present the spatial distribution of the number of kindergartens (per million persons) at city scale. Next, in section 3 we investigate the influential factors and their interaction effects on the spatial distribution of kindergartens using the geographical detector method. Lastly, we provide policy suggestions in section 4.

## 2. Methods and data sources

In this paper, we first employ Pearson correlation to establish basic linear relationships. However, recognizing that socio-economic data often violates normality assumptions, we supplement this with the Grey relational degree method, which is more robust for non-linear and small-sample data. Finally, to directly address our core interest in spatial heterogeneity and factor interactions, we utilize the Geographical Detector model, which is specifically designed for this purpose and avoids multicollinearity issues.

### 2.1. Pearson correlation coefficient

The Pearson correlation coefficient is commonly applied to measure linear correlation between two variables. It is defined as the ratio of covariance of two variables to the product of their standard deviations. Specifically, it reads as


$$\rho =\frac{\Sigma_{i=\text{1}}^{n}(x_{i}-\overset{―}{x})(y_{i}-\overset{―}{y})}{\sqrt{\Sigma_{i=\text{1}}^{n}{\left(x_{i}-\overset{―}{x}\right)}^{\text{2}}}\sqrt{\Sigma_{i=\text{1}}^{n}{\left(y_{i}-\overset{―}{y}\right)}^{\text{2}}}},$$ (1)

where ρ is the coefficient ranging from −1–1. $\overset{―}{x}$ and $\overset{―}{y}$ represent the average values of variable *x* and *y*, respectively. We apply the Pearson correlation coefficient to measure the linear relationships between socioeconomic factors and number of kindergartens.

### 2.2. Grey relational degree

The Pearson correlation coefficient, as an important statistical method, also has basic requirements despite being used in many empirical studies. For example, it requires that statistical data must satisfy some specific distribution, like normal distribution. Likewise, the linear regression approach, also commonly applied in literature, assumes that the Gauss-Markov theorem should be satisfied; otherwise, biased conclusions may result. To overcome these shortcomings, the Grey relational degree approach was first proposed by Deng [9]; it is used to measure the quantitative correlation between two variables without such prior requirements. It also has a merit in that it is able to work with small sample sizes of irregular data. Hence, it has gained popularity in measuring the correlations in existing literature. This approach is introduced below.

Suppose a reference sequence ${\text{x}}_{\text{0}}(i)$ and a set of comparison sequence ${\text{x}}_{\text{j}}(i)$. In our study, the former is treated as the number of kindergartens, and the latter denotes a series of socioeconomic factors. Thus, the grey relational degree coefficient between each factor and the dependent variable is computed as


$$\xi i(i)=\frac{\frac{min}{j}\frac{min}{i}\left|x_{\text{0}}(i)-x_{j}(i)\right|+\rho \frac{max}{j}\frac{max}{i}\left|x_{\text{0}}(i)-x_{j}(i)\right|}{\left|x_{\text{0}}(i)-x_{j}(i)\right|+\rho \frac{max}{j}\frac{max}{i}\left|x_{\text{0}}(i)-x_{j}(i)\right|}$$ (2)

where ξ denotes the grey relational degree. $\frac{max}{j}\frac{max}{i}\left|x_{\text{0}}(i)-x_{j}(i)\right|$ and $\frac{max}{j}\frac{max}{i}\left|x_{\text{0}}(i)-x_{j}(i)\right|$ are the smallest and biggest absolute difference values, respectively. ρ is a key parameter introduced in the equation to differentiate the degree of proximity of $x_{\text{0}}$ and $x_{j}$. It ranges from 0 to 1. Empirically, it takes 0.5.

Then we can calculate the grey relational degree value in accordance with the following equation


$$\gamma_{\text{i}}=\frac{\text{1}}{n}\Sigma_{i=\text{1}}^{n}\xi j(i),$$ (3)

where γ represents the grey relational degree between ${\text{x}}_{\text{0}}$ and ${\text{x}}_{j}$, and is restricted to the interval [0, 1]. Different from the Pearson correlation coefficient, the grey relational degree can merely measure how close $x_{j}$ is to $x_{\text{0}}$, rather than detect the positive or negative direction. In this study we adopt it to calculate the grey relational coefficient between each factor and the number of kindergartens.

### 2.3. Geographical detector analysis

In regard to spatial stratified heterogeneity, Wang et al. [10] proposed a novel spatial variation approach, namely, the geographical detector model, to detect and measure the spatial heterogeneity of a geo-referenced variable. The approach has two main advantages. One is to quantify the association between pair geo-referenced variables if it assumes that their stratified heterogeneity tends to be similar without a linearity prerequisite. In essence, it is used to evaluate the spatial similarity of two geo-referenced date sets in spatial distribution. The other is that it can examine the interaction effect between two independent variables on the dependent variable. Due to its merits, a growing number of applications of this approach are found in the literature, e.g., welfare homes [11], the Covid-19 epidemic [12], housing rents [13], and innovation productivity [14]. In this study, we apply this method, specifically both factor detector and interaction detector to identify and quantify the correlations between socioeconomic factors and number of kindergartens of 323 Chinese cities.

The factor detector is used to measure the relationship between factor *X* (influencing factors) and the dependent variable, *Y* (number of kindergartens). It can be expressed as


$$q=\text{1}-\frac{\Sigma_{\text{h}=\text{1}}^{L}N_{h}\sigma_{h}^{\text{2}}}{N\sigma^{\text{2}}}=\text{1}-\frac{SSW}{SST}$$ (4)

$$\text{SSW}=\Sigma_{\text{h}=\text{1}}^{L}N_{h}\sigma_{h}^{\text{2}}$$ (5)

$$SST=N\sigma^{\text{2}},$$ (6)

where *h(1, 2, …, L)* represents the number of subregions of factor *X*. *N*_*h*_ and *N* represent the number of samples in subregion *h* and the whole study area, respectively. Accordingly, $\sigma_{h}$ and σ denote the variance of samples in subregion *h* (SSW) and the total variance (SST), respectively. *q* statistic denotes the geographical detector factor value, which ranges from 0 to 1. The larger *q* value, the stronger explanatory power, and vice versa. Specifically, the *q* value can be explained by the explanatory power of factor *X* on the dependent variable Y. In other words, it implies that factor X explains 100*q^%^ of *Y*.

The concept of the interaction detector considers the interaction impact of two different factors, e.g., X1 and X2, on the dependent variable, Y. q(X1) and q(X2) denote the q values of factor X1 and X2, respectively according to Eq. 2. Moreover, a new factor strata can be generated by overlaying the factor strata X1 and X2, labeled as x1∩x2, where the sign ∩ is the interaction. Next, the q value of the interaction effect of x1∩x2 (q(x1∩x2)) can be computed. The results of the interaction effect can be classified into 5 categories, which are summarized in Table 1.

**Table 1:** Interaction categories of two factors and the types of the interaction relationship.

Type	Description	Interaction effect
1	q(x1∩x2) <Min[q(x1), q(x2)]	Weaken; univariate
2	Min(q(x1), q(x2))< q(x1∩x2) <Max[q(x1), q(x2)]	Weaken; univariate
3	q(x1∩x2)> Max[q(x1), q(x2)]	Enhanced, bivariate
4	q(x1∩x2) = q(x1) + q(x2)	Independent
5	q(x1∩x2) > q(x1) + q(x2)	Nonlinearly enhanced

### 2.4. Variable selection and data sources

In this study, we intend to demonstrate the spatial distribution of the kindergartens and their influencing factors in China. To this end, we have chosen a total of 323 prefecture-level cities and four municipalities (namely, Beijing, Tianjin, Shanghai, and Chongqing). Based on our above introduction, we have considered 4 categories of factors, i.e., population factors, education factors, urbanization factors, and economic factors. We seek to explore whether they affect the spatial distribution of kindergartens in Chinese cities.

The first category, population factors, includes three variables, namely, the proportion of marriageable population (marry), proportion of population in high school or above (hsr), and proportion of immigrants (immi). Marriageable population is considered as a strong indicator of the total fertility rate (TFR) [15], which ostensibly leads to the relatively high need for kindergartens. Parents’ education background and education level have shown to be associated with their expectations of their children’s education and academic performance [16,17]. We therefore assume that parents with a high education level have high expectations for their children’s preschool education, resulting in demand for kindergartens. Immigrants indeed have different needs compared with non-immigrants in terms of preschool education; therefore, the variation of the proportion of immigrants is expected to be associated with the distribution of the kindergartens.

The second category, education factors, is composed of the average years of education (edu) and the proportion of the population employed in the education sector (teach). Biddle’s [18] study shows that education level of household members is positively related to children’s preschool attendance. Barnett & Jung [19] illustrate that parents’ education has a strong impact on children’s preschool participation rate; this may be because parents with different education levels show different expectations of their children’s preschool performance and academic skills. This factor is likely to influence the decision whether parents place importance on children’s preschool education [16]. Therefore, it is safe to assume that average years of parental education in different cities should have different needs for preschool education. As mentioned above, the Chinese government has invested considerably in preschool education to make preschool education inclusive. We assume that more people employed in the preschool education sector should be related to the increase of kindergartens.

The third category, urbanization factors, consists of urbanization rate (urban) and total sales of commercial residential buildings per person (est). The literature has proved that the urbanization process is closely associated with the Total Fertility Rate (TFR), which is especially the case in China during the past 40 years [20,21]. This would certainly influence the demand and distribution of kindergartens in different cities. We also select total sales of commercial residential buildings per person as an important predictor, because different local taxes that could be used to build kindergartens are largely coming from real estate sales in Chinese cities. Other evidence indicates that the cost of raising children is almost always associated with housing [22]. In fact, housing characteristics have been shown to influence people’s fertility intentions [23,24,25]. In a general sense, Chinese couples used to have children after purchasing houses or apartments [26]. The total sales of commercial residential buildings per person may positively be associated with the need for kindergartens.

The fourth category, economic factors, includes three variables: income per capita (inc), per capita income of urban residents (uinc), and per capita education expenditure (edux). Income per capita indicates the ability to pay for preschool education, which is important in the demand for kindergartens. As mentioned previously, kindergarten education is not a compulsory education so parents have to pay for their children to attend; this cost is even higher if the kindergarten is privately funded. Nevertheless, since 2018, China has focused on establishing more affordable kindergartens through increased public education expenditure [27]. Such public expenditure on education is expected to have an impact on the construction and distribution of kindergartens.

All explanatory variables in the 4 categories were derived from the 2023 edition of the China City Statistical Yearbook. The web crawler technology was applied to confirm the location information and number of kindergartens from the Baidu Map API service in March of 2022. Specifically, Kindergarten location data (POIs) were collected using the Baidu Maps Place API in March 2022. We the keyword “幼儿园” (kindergarten) within the administrative boundary, targeting 327 cities, including 323 prefecture-level cities and 4 municipalities to capture two key data points, namely, the location information of kindergartens. Queries were iterated using a 2 km grid to ensure full spatial coverage. The returned JSON data included name, address, latitude/longitude, and POI type. After removing duplicates (based on exact name + coordinate match), we obtained more than 200 thousand kindergarten records. City-level counts were aggregated using a spatial join with 2021 administrative boundary shapefiles from the National Catalogue Service for Geographic Information. API key and full raw POI data compliance with Baidu’s Terms of Service. Besides, the data collection and analysis complied with terms and conditions of the source of data. The descriptive statistics for the variables used in this research are summarized in Table 2.

**Table 2:** Descriptive statistics for variables involved in the analysis.

Variable	N	Mean	p25	p50	S.D.	Min	Max
*Kinderpc*	327	13.98	9.768	13.46	5.937	1.673	34.84
*Marry*	327	0.298	0.267	0.294	0.0440	0.208	0.476
*Hsr*	327	0.215	0.158	0.199	0.0820	0.0450	0.527
*immi*	327	0.107	0.0350	0.0610	0.118	0.00800	0.799
*edu*	327	8.812	8.300	8.830	0.999	3.890	11.71
*teach*	327	0.0240	0.0190	0.0220	0.00700	0.0100	0.0530
*urban*	327	29.57	18.39	25.82	15.54	6.490	86.23
*est*	327	0.762	0.342	0.595	0.589	0.00500	4.764
*inc*	327	11610	8360	10555	4987	3786	33935
*uinc*	327	16858	14333	15620	4298	8260	35690
*edux*	327	791.8	576.0	704.4	344.6	317.1	2949

Using above mentioned methods, we first present the visual spatial distribution of kindergartens in Section 3.1.

## 3. Empirical results and discussion

### 3.1. Spatial distribution of kindergartens

The spatial distribution of kindergartens is demonstrated in three figures through ArcGIS 10.5.

**Fig 1:**
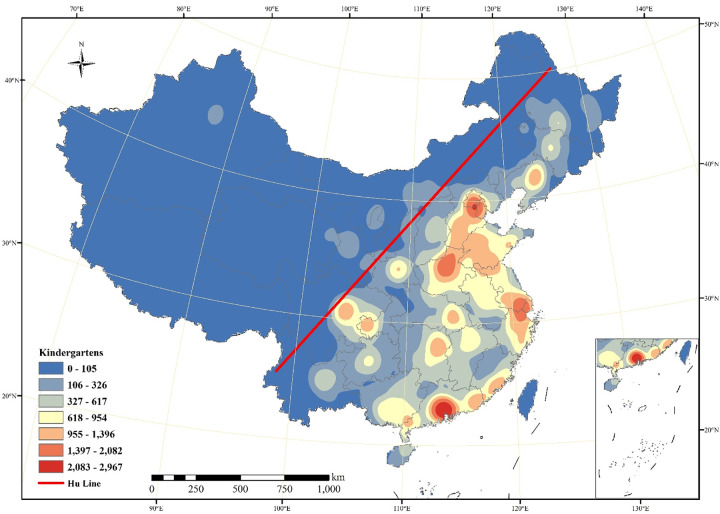
Kernel density estimates for number of kindergartens in 323 Chinese cities.

**Fig 2:**
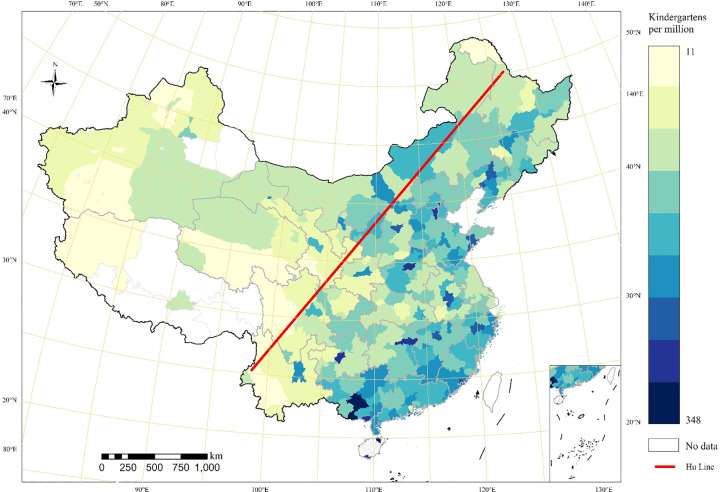
Spatial distribution of number of kindergartens per million persons by cities.

**Fig 3:**
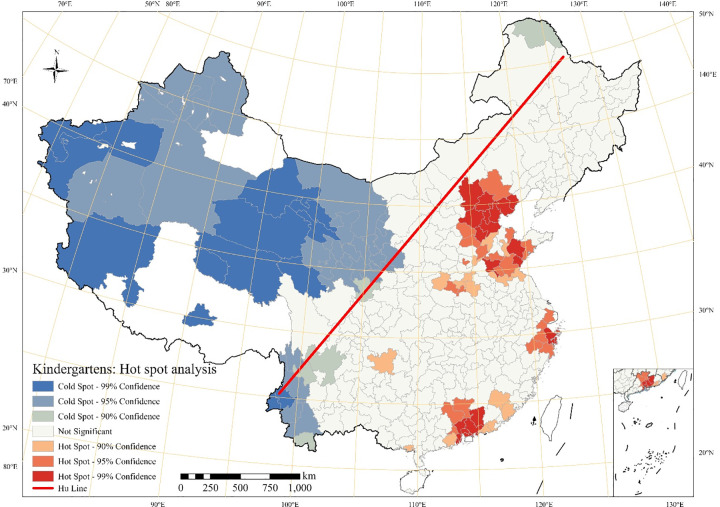
Hotspot analysis of the number of kindergartens by cities.

Specifically, we first display the kernel density estimates for kindergartens of Chinese cities in Fig 1; second, we normalize the number of the kindergartens per million persons to eliminate the population and city size in Fig 2; third, we perform a Hotspot analysis to illustrate to what extent the kindergartens are clustered in Fig 3. The spatial distribution shown reflects the absolute provision of kindergarten infrastructure (Fig 1) and its relationship to total population size (Fig 2), which is apparently influenced by both economic capacity and demographic structure.

In Fig 1, we can observe that the highest density areas are generally with high economic development levels and urbanization rates and population aggregation, which include the Bohai Economic Rim, the Yangtze River Delta region and the Pearl River Delta region. There are also high values in part of the Sichuan-Chongqing urban agglomeration, the Beibu Gulf Economic Zone, the middle reaches of the Yangtze River urban agglomeration and Fujian Province. Correspondently, the regions with the lowest values are mostly located in northwest China, parts of Yunnan, Guizhou, Sichuan provinces as well as much of the northeastern region. The exception is the Liaodong Peninsula, where the economic development level is the highest within the northeastern region. We can conclude that the low values areas are generally associated with relatively low economic growth and less population. In general, the Hu Huanyong population line (also known as the Hu Line; for details, see [28]) can be seen as the dividing line between the high- and low-density regions of the distribution of kindergartens. Although we do see some regions located in the eastern part of the Hu Line with low values, these regions are nearly all mountainous areas with relatively poor natural conditions and low population density, e.g., Jiangxi province and the area of Wuyi Mountains. Therefore, it is safe to say that the distribution of kindergartens is associated with the distribution of the population.

Fig 2 depicts relatively considerable changes after the normalization of population size in terms of the spatial distribution of kindergartens. Compared with Fig 1, the normalized high values are scattered rather than aggregated in general. Specifically, the low values are mainly distributed in Tibet Autonomous Region, most of Xinjiang Uygur Autonomous Region, Yunnan, Sichuan, Qinghai and Gansu Province where economic development is relatively low compared to the Eastern regions. Judging by the number of kindergartens, the Hu Line serves as an important and clear boundary dividing the number and distribution of kindergartens into two parts, the western inland cities and the eastern coastal cities. The cities with more people and higher density are all distributed on the right side of the Hu Line. Specifically, four regions with the highest density are mainly the Bohai Economic Rim, Yangtze River Delta and Pearl River Delta, which are more economically developed, and Henan Province, with its high population density. The cities with the second largest number are some provincial capitals, such as Changsha, Wuhan, Chengdu, and Shenyang. In the western region, especially the area to the left of the Hu Line, the number of kindergartens is relatively low. Moreover, the total number of kindergartens shows a decreasing trend from east to west. However, the spatial distribution of the number of kindergartens per million people (Fig 2) differs from the number of kindergartens (Fig 1). The spatial distribution is more scattered and there are areas with a large number of kindergartens per million people in the southeast, northwest, and central China. Among them, Fangchenggang, Nanning, Beihai in Guangxi Province in the southwest, and Haikou and Sanya City in China’s most southern province of Hainan have the largest number of kindergartens per million people, more than 300. The cities with the highest number of kindergartens per million people in the eastern provinces are Xiamen in Fujian Province, Langfang in Hebei Province, Shenyang and Panjin in Liaoning Province, Zhuhai in Guangdong Province, Jinhua in Zhejiang Province, and Qingdao in Shandong Province. The central provinces with the largest number of kindergartens per million people are basically all provincial capitals, e.g., Zhengzhou, Guiyang, Changsha, Shenyang, Nanchang, Hefei, and Taiyuan. Yinchuan and Xining provincial capital cities, located on the left side of the Hu Line, also have relatively large numbers of kindergartens per million people. In addition, it is noteworthy that the number of kindergartens per million people in the middle part of Yangtze River Delta region is not high relative to its south and north, which may be related to the age structure of the population in these regions, specifically, the most aging region in China.

To better identify the dense and sparse areas in terms of the distribution of kindergartens, we apply Hot Spot Analysis and display it in Fig 3. The hot spots of the kindergartens are mainly aggregated in the Bohai Economic Rim, especially the Jing-jin-ji region, the Yangtze River Delta region, and the Pearl River Delta region. In general, these regions are clustered with relatively larger populations, higher economic levels and higher urbanization rates. The cold spot areas are concentrated mainly in the Xinjiang Uygur Autonomous Region, Qinghai province, Ningxia Hui Autonomous Region, northwest Tibet Autonomous region, and part of Gansu and Shanxi provinces. Additionally, Southwest China, particularly in Yunnan, part of Sichuan and Guizhou province also depict a cold spot concentration. The implication here is that there should be a relatively strong connection between number of kindergartens and high economic development level.

Having established clear spatial patterns, we now employ statistical methods to identify the socioeconomic factors driving this distribution.

### 3.2. Results of Pearson correlation coefficients and grey relational degrees

Before we conduct the geographical detector analysis, we apply the Pearson correlation coefficient approach as a benchmark to measure the linear relationships between socioeconomic factors and the dependent variable, e.g., number of kindergartens per million persons. The results of the Pearson correlation coefficients are summarized in Table 2. We observe that all 10 explanatory variables are statistically significant at least at a 10% significance level, indicating that they are all important factors affecting the number of kindergartens. The Pearson correlation coefficients of the 10 variables in descending order are as follows: *edu* (0.560)> *inc* (0.468)*> hsr* (0.440)*> uinc* (0.423)*> est* (0.381)*> teach* (0.360)*> immi* (0.331)*> urban* (0.276)*> marry* (0.113)*> edux* (−0.097). We find that the variable *edu* with the highest coefficient is the foremost factor, confirming that average years of education determines number of kindergartens. In addition, these 10 factors are positively correlated with the dependent variable, except the *edux* variable. The main reason for this is that they are not normally distributed variables after a normality test is performed. In other words, linearity is a prior restriction for the relationship. Similarly, we also perform such tests for the other variables and find that the hypothesis of normal distribution is strongly rejected. We can conclude that the Pearson correlation coefficient approach may not suitable for our study.

To overcome this shortcoming, we employ the grey relational degree to quantify the geometric relationships and the correlation strengths between socioeconomic factors and number of kindergartens (Table 3). On the one hand, we observe that the grey relational degree value of each factor is much larger than the Pearson correlation coefficient. It may mean that the Pearson correlation coefficient approach could underestimate the association strengths between not normally distributed variables. On the other hand, the values in descending order also change as follows: *edu* (0.917)*> marry* (0.906)*> uinc* (0.871)*> teach* (0.883)*> urban* (0.873)*> inc* (0.871)*> hsr* (0.866)*> est* (0.857)*> edux* (0.839)*> immi* (0.815). Once again, the *edu* variable with the highest value is also treated as the foremost factor in the grey relational degree analysis. The *edux* variable also has a high value, indicating an important role in number of kindergartens. However, like the Pearson correlation coefficient approach, the grey relational degree method does not consider underlying spatial stratified heterogeneity, which may lead to biased conclusions.

**Table 3:** Results of Pearson correlation coefficients and grey relational degrees.

Variable	Population factors			Education factors		Urbanization factors		Economic factors		
	*marry*	*hsr*	*immi*	*edu*	*teach*	*urban*	*est*	*inc*	*uinc*	*Edux*
Pearson	0.113 [0.042]	0.440 [0.000]	0.331 [0.000]	0.560 [0.000]	0.360 [0.000]	0.276 [0.000]	0.381 [0.000]	0.468 [0.000]	0.423 [0.000]	−0.097 [0.079]
Grey	0.906	0.866	0.815	0.917	0.883	0.873	0.857	0.871	0.901	0.839

*Note*: p values in brackets.

### 3.3. Results of geographical detector analysis

The geographical detector approach has a prerequisite that the factor variables should be discrete variables. When it is a continuous variable, it should take discretization transformation by using classification suggested by Wang et al. [10]. Empirically, there are three types of data classification methods, namely, quantile (Qu), equal intervals (Eq), and natural breaks (Na). Since the result of geographical detector analysis is sensitive to both the classification method and the number of classes, which determines the explanatory powers of socioeconomic factors, for the purpose of the most powerful explanation, we adopt an optimal discretization approach to classify each socioeconomic factor and obtain the most suitable number of classes. Results are summarized in Table 4.

**Table 4:** Results of optimal discretization of socioeconomic factors.

variable	*marry*	*hsr*	*immi*	*edu*	*teach*	*urban*	*est*	*inc*	*uinc*	*edux*
Method	Qu	Eq	Qu	Na	Qu	Na	Na	Qu	Qu	Qu
Number	8	8	6	7	7	8	8	5	7	8

When compared with the Pearson correlation coefficients and the grey relational degree, the geographical factor detector method has many advantages in empirical studies. One is that it can capture the spatial heterogeneity of the distribution of the number of kindergartens. The second is that we can identify and quantify the socioeconomic factors of the number of kindergartens under the assumption that the pair variables are associated if their spatial stratified heterogeneity tends to be consistent. The third is that it can avoid the problem of multicollinearity among the explanatory variables that regression models typically suffer from. In addition, it does not impose a prior assumption that all variables are independently and identically distributed, which is usually required in regression analysis. The last merit is that it is able to investigate the interaction effects of two socioeconomic factors on the number of kindergartens and identify if the interaction effect of two socioeconomic factors weakens or enhances the impact on the number of kindergartens.

**Fig 4:**
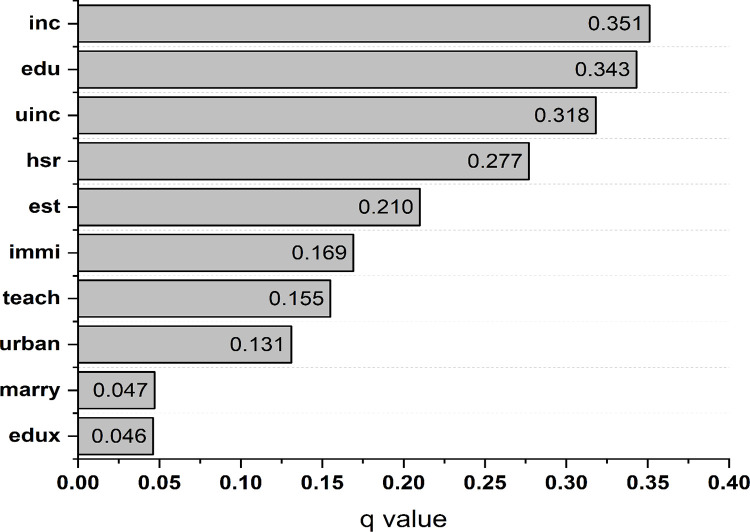
q values of explanatory variables using factor detector analysis.

The results of factor detector analysis are plotted in Fig 4. The q values in descending order are: *inc* (0.351)*> edu* (0.343)*> uinc* (0.318)*> hsr* (0.277)*> est* (0.210)*> immi* (0.169)*> teach* (0.155)*> urban* (0.131)*> marry* (0.047)*> edux* (0.046). Overall factor detector analysis results are similar to those of the Pearson correlation coefficients, but the orders change slightly. For example, the *inc* variable is the foremost factor of the number of kindergartens in the factor detector analysis, followed by the *edu* variable. In contrast, in the Pearson coefficients, the *edu* variable has the highest value, followed by the *inc* variable. To better display the differences in the results using the three methods, we summarize the orders of the impacts of the socioeconomic factors on number of kindergartens in Table 5.

**Table 5:** Orders of the correlation values of socioeconomic factors.

Method	inc	edu	uinc	hsr	est	immi	teach	urban	marry	edux
q factor	1	2	3	4	5	6	7	8	9	10
Pearson	2	1	4	3	5	7	6	8	9	10
Grey	6	1	3	7	8	10	4	5	2	9

We can verify that factor detector analysis is able to correct the overestimates of the Pearson correlation coefficients method when considering spatial stratified heterogeneity.

We observe that the q values of three variables are higher than 0.3, namely *inc*, *edu*, and *uinc*. Specifically, the *inc* variable has the highest q value, followed by *edu* and *unci*. Since *inc* and *uinc* are highly correlated, we can verify income per capita and average years of education as foremost determinants of the number of kindergartens of Chinese cities. Income per capita indeed plays a decisive role in the establishment of kindergartens. On the one hand, this is because cities with higher per capita income usually have more money to invest in preschool education; moreover, they also have diversified demand for different types or levels of kindergartens, notably private capital funded kindergartens. Parents in these cities are more likely to send their children to kindergarten to access better preschool education. On the other hand, these relatively rich cities are better suited to provide fiscal support for the demand of kindergartens. What’s more, cities with higher education levels tend to prioritize education, including preschool, since it is widely regarded as a fundamental part of the education system.

The proportion of population in high school or above is also an important factor affecting the distribution of number of kindergartens in Chinese cities, since the q value of the *hsr* variable reaches nearly 0.3 (0.277), just behind per capita income and education. Next, the *immi* variable has a q value of 0.210, indicating that the total sales of commercial residential buildings per person (*est*) is regarded as a determinant of number of kindergartens. In general, the total sales of commercial residential buildings are determined by both local citizens and immigrants. Large numbers of migrants flow into cities, leading to an increase in total sales of commercial residential buildings and consequent higher demand for more kindergartens. Hence, the *immi* variable is also a key factor and its q value reaches 0.169. The remaining four variables, namely, *teach*, *urban*, *marry* and *edux* are also statistically significant determinants of the number of kindergartens. Importantly, the *edux* variable has the least q value since the spatial distribution is the least similar to number of kindergartens. It also implies no linear relationship between education expenditure and number of kindergartens. There may be two reasons for this. First, private capital funded kindergartens also play an important role in these cities since they can meet the diversified demand for multiple types of kindergartens. Second, the underlying fact is that preschool education is not yet compulsory in education systems.

**Fig 5:**
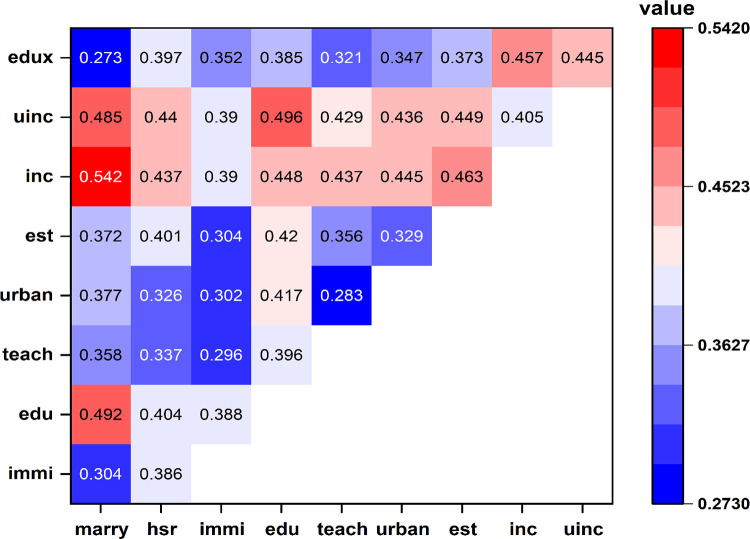
Results of interaction detector analysis.

From our factor detector analysis, we can observe the extent to which a single factor can explain the spatial distribution of number of kindergartens in Chinese cities. However, it is always the case that more than one driving factor is affecting the dependent variable. The interaction effects of pair factors are given in Fig 5 using the interaction detector.

Fig 5 displays the q-values of 44 pairs of interaction terms among 10 explanatory variables. They are all significant at least at a 10% level. We find that interaction between two factors has a stronger effect on the number of kindergartens. In other words, the dependent variable, kindergartens per million persons, is likely to be strengthened by the interaction effect of two factors rather than one. Specifically, the explanatory power of the interaction is enhanced when compared with one individual factor. Together with the results of factor detector analysis, we can conclude that *edu*, *unic* and *inc* are the most important factors when they interact with other factors. Moreover, this also means that the variation of any explanatory variable may cause relatively large changes in the distribution of the number of kindergartens. What is even more interesting is that the influence of per capita education expenditure (*edux*) on number of kindergartens per million persons is relatively weak but becomes much stronger when interacting with other factors, e.g., the *marry* variable. This, to some extent, confirms that the explanatory power of a univariate weakens the degree of interpretation. On the contrary, the *urban*, *teach* and *immi* variables are comparatively less important factors in the interaction effect and reflects the fact that, although urbanization can be seen as the influencing factor in the number of kindergartens, its explanatory power is relatively weak. The reason is because the Chinese government has been responsible for the establishment of kindergartens in rural and less developed regions such as in Northwest China, therefore, kindergartens in these underdeveloped areas may be relatively adequate. Moreover, due to the Chinese household registration system, people who do not obtain a local household registration are usually excluded from the local public welfare and urban services, e.g., publicly funded kindergartens in this study. This also explains why the proportion of immigrants (*immi*) is less important to the kindergartens per million persons.

With regard to the interaction effects, we pay special attention to two variables, namely, *uinc* and *inc* because both are relatively prominent and can enhance the effects of the other variables. In particular, the interactions between *marry* and three other variables, namely, *uinc*, *inc* and *edu*, can explain the number of kindergartens per million persons to a very large extent, although *marry* alone plays a relatively weak role in the distribution of kindergartens. This is reasonable because having babies before marriage in China is very rare, even if couples are wealthy or highly educated; also, women who have babies out of wedlock may still be considered as the family shame in many places in China [29]. The interactive effects of two education related variables, *hsr* and *edu* and two income related variables, both *uinc* and *inc* indicate that people with higher incomes tend to consider putting more money into preschool education. It has been proved that parents’ education level is positively associated with children’s preschool participation [30,31]. On the contrary, low-income parents may not even be able to afford their children’s preschool education fees, especially considering that preschool education is not free in China. In fact, it is likely that those with higher education level or accepting more education have higher incomes and vice versa. Therefore, these two variables stimulate the demand for kindergartens, notably diversified types of kindergartens. The *teach* variable is also enhanced by *uinc* and *inc*. It may be the case that a higher proportion of the population employed in the education sector with high income can indicate the investment of education facilities, which includes the kindergartens per million people in this research. The *urban* variable will significantly determine the number of kindergartens per million people when it interacts with *uinc* and *inc*. The demand for preschool education is still largely emerging from urban populations even though the Chinese government has made large investments in rural areas over the past decade. Nevertheless, areas with higher income and urbanization rate will invest more. The *est* variable illustrates that the local tax contributes to investment in the education sector. Public kindergartens are largely reliant on the local tax while the local tax system is largely garnered from total sales of commercial residential buildings in China [32]. Moreover, as a result, places with high income from sales of commercial buildings will enhance the establishment of kindergartens as part of their preschool education system.

## 4. Conclusions and policy implications

Hong et al. [33] have predicted a relatively higher demand for preschool education in the next 15 years compared to the available resources in 2019 even though the seemingly opposite is argued: that children enrolled in kindergarten will decline from 2021 to 2035. The contradictions are, on the one hand, the population of children aged 3–6 years will drop from 65.12 million in 2021 to 42.66 million in 2035 (decline from 40.34 million to 29.71 million in urban while decrease from 24.78 million in 2021 to 12.95 million in rural respectively). On the other hand, the birthrate will drop from 7.52‰ in 2021 to less than 5.0‰ in 2035. Notably, the number of annual births has nearly halved from over 18 million in 2016 to just 9.02 million in 2023. This decline will directly translate into a shrinking school-age population (typically defined as children aged 6–18) with a 5- or 6-years lag. Additionally, the current preschool education enrollment rate is relatively low, 89.7% in 2022 compared to some European countries such as France, Ireland, Portugal and the UK where the preschool education enrollment rate is already 100% [34]. Considering the large population base in China, a small promotion in percentage would increase a large demand for kindergartens.

This article has analyzed the numbers and the distribution of kindergartens and their socioeconomic impact factors. The results of the kernel density estimation demonstrate that the number of kindergartens is highly aggregated in eastern coastal cities. The spatial distribution of kindergartens is more or less associated with the distribution of population and income. Together with the Hotspot analysis result, the spatial distribution of number of kindergartens per million persons reveals that a high economic development level is essential for the development of kindergartens. We have used the Pearson correlation coefficients and Grey relational degrees to emphasize the significance of the average years of education to the kindergartens per million persons. In the results of the geographical detector analysis, both per capita income of urban residents and income per capita can enhance the explanatory power of other variables, notably when considering interaction effects. This means that it is important for the government to increase income in order to promote preschool education. We have also identified 10 influencing factors that impact on the kindergartens per million persons. The influencing factors can be summarized into four types, namely, educational, urbanization, economic, and population factors. Policy makers should include these factors into their policy making for preschool education in China.

In the past 10 years the Chinese government has made great strides in narrowing the opportunity gaps in preschool education in different places and forms. However, gaps remain between eastern coastal cities and inland cities, between rural and urban, and between privately operated and publicly funded kindergartens. The results of this study show that both total number and average number of kindergartens per million people are much higher in eastern coastal cities, and are aggregated in the most developed regions, e.g., the Bohai Economic Rim, the Yangtze River Delta, and the Pearl River Delta. In these areas, fiscal expenditure and private capital investment in preschool education are adequate, and it is more likely that these regions would benefit from market-oriented policies on preschool education. Nevertheless, cities in the west and middle areas may still rely on fiscal transfer from central government due to poor economic development level and population loss. We advocate that the government should designate the public kindergartens as compulsory education, where parents only need to pay for meals and teaching materials. Considering that the labor cost is relatively high in east and coastal areas, it may be beneficial to conduct a pilot scheme in the central and western regions.

Although efforts have also been made to support kindergartens in rural areas, they are still insufficient. This is because children are scattered across vast mountainous or arid regions, making centralized service delivery difficult and expensive. Besides, rapid urbanization has led to an out-migration of young working-age adults to cities, leaving behind “left-behind children” in the care of grandparents. This shrinks the potential kindergarten population in many villages, making it economically unviable to maintain a standard facility. Moreover, China’s fiscal system is highly decentralized. Rural townships and counties have a very weak tax base and rely heavily on transfers from higher levels of government. Funding for building and, crucially, operating kindergartens (teacher salaries, utilities, maintenance) is scarce. Planning mandates from the central government often come without sufficient earmarked funding, leading to implementation gaps. Many remote villages lack reliable running water, modern sanitation, or stable electricity, which are basic prerequisites for a licensed kindergarten. Building a new kindergarten first requires massive investment in this underlying infrastructure. In rural China, the top-down planning model struggles against geographic and fiscal realities. The result is a chronic shortage of quality provision. Planning here should be adaptive, flexible, and heavily subsidized. Initiatives like “One Village One Kindergarten” represent a bottom-up supplementation of the top-down system, focusing on accessibility over ideal standards. It can be said that the spatial distribution of kindergartens reflects broader socioeconomic inequalities. Therefore, to overcome socioeconomic inequalities can be an important step to achieve a more equitable distribution. It is generally considered China’s urban planning apparatus has the technical capacity to distribute kindergartens efficiently, however, achieving equitable distribution requires overcoming deep-seated issues of fiscal decentralization.

We can observe from geographical detector that urbanization rate, proportion of rural-urban migrants, and total sales of commercial residential buildings are all essential factors to the development of preschool education in urban areas. The implication here is that the regional inequalities of preschool education will increase if no effort is made in the future in rural areas; In fact, in many developing countries such as Peru, Vietnam and India where the imbalanced and disadvantaged economic development and the uneven distribution of resources across regions would almost always lead to a rural-urban divide in terms of enrollment rates, the distribution of private services and the quantity and quality in preschool education (Betancur et a., 2024). Therefore, the development of preschool education in rural areas in developing countries may still have to be dependent on the fiscal expenditure of the government. Meanwhile, Woodhead et al. (2009)[35] summarize that the reason why such rural-urban divide existed in preschool education is that urban families are more capable of paying. However, it is unrealistic to raise the payment capacity to preschool education in rural areas in a short time especially in developing countries. As early as 2010 the State Council outlined “the National Medium- and Long-term Education Reform and Development Plan (2010-2020),” which established a system of preschool education led by government, with social participation, and both public and private enterprise.

Based on the insights from the interaction effects analysis, it is evident that the spatial distribution of kindergartens is not merely influenced by individual socioeconomic factors in isolation, but rather by their synergistic interactions. For instance, the interaction between per capita income (*inc*) and average years of education (*edu*) significantly enhances explanatory power, suggesting that policies aimed at simultaneously raising household income and educational attainment could have a multiplicative effect on improving kindergarten accessibility and quality. Similarly, the interaction between urbanization rate (*urban*) and income variables underscores the importance of coordinated regional development strategies that integrate economic growth with urban planning to ensure equitable distribution of preschool education resources. These findings imply that policymakers should adopt a multi-dimensional approach, designing interventions that leverage these interactive effects—such as combining fiscal incentives for kindergarten construction in low-income areas with programs that boost local education levels and urban infrastructure—to create more targeted, effective, and sustainable policies for promoting accessible and high-quality preschool education across diverse socioeconomic contexts.

Although in “the report on the work of the government 2020”, the central authority asserted that one of its significant tasks is to strongly support inclusive preschool education and help to establish private kindergartens, which this study has shown are still badly needed. By saying inclusive preschool education, we refer to a system of early childhood education and care that is universally accessible, financially affordable and quality guaranteed, and meets basic needs for all children aged 3–6, with a special focus on those from disadvantaged backgrounds (not solely about including children with disabilities but a broader socioeconomic policy aimed at making early childhood education a public good). While significant challenges in funding, teacher quality, and implementation persist, the commitment to its principles and objectives remains a central pillar of China’s modern education agenda. In fact, most private kindergartens are purely a trade investment and are far from “inclusive”. Therefore, robust financial support from government would guarantee the promotion and development of inclusive kindergartens. However, at present, China’s financial investment is largely directed to the number and the quality of public kindergartens; investment in private kindergartens is quite small. This is quite different from some developed countries such as UK, USA, Germany, and the Netherlands where the private sectors are directed by relevant policies to play a more significant role in the preschool education system (Zhou and Lu, 2024)[36] . This means establishing a clear investment system and improving it, along with increasing financial investment in private kindergartens is an important step towards turning private kindergartens into inclusive ones. In a more detailed way, government funding should subsidize mandatory training programs for private kindergarten teachers and principals on inclusive education practices. This can include funding for expert trainers, creating resource networks, and providing stipends to teachers who undergo certification. And the subsidies should also be granted to eligible families (e.g., low-income, migrant, or families with children with disabilities) in the form of vouchers that can be used at any government-approved private kindergarten. Alternatively, the government can pay the kindergarten a subsidy for each enrolled child from a target group.

There are limitations due to the data we acquire. First, without data on the target age group (3–6 years), the maps in this paper should be interpreted as showing potential access and general investment levels rather than a precise measure of supply-demand balance. Second, our study identifies the macroeconomic and spatial supply of kindergarten infrastructure, it does not account for qualitative disparities. In the same way, a high density of kindergartens does not necessarily equate to high-quality, accessible, or sufficient preschool education, as institutions can vary greatly in their capacity and resources. Last, the subsequent studies could incorporate metrics of quality and capacity, such as the average student-teacher ratios per city, actual enrollment numbers versus capacity or even physical infrastructure indices.
